# No robust relation between larger cities and depression

**DOI:** 10.1073/pnas.2118943118

**Published:** 2022-01-05

**Authors:** Karoline B. S. Huth, Adam Finnemann, Maarten W. J. van den Ende, Peter M. A. Sloot

**Affiliations:** ^a^Department of Psychology, University of Amsterdam, 1001 NK Amsterdam, The Netherlands;; ^b^Centre for Urban Mental Health, University of Amsterdam, 1012 GC Amsterdam, The Netherlands;; ^c^Department of Psychiatry, Amsterdam University Medical Center, 1105 AZ Amsterdam, The Netherlands;; ^d^Computational Science Lab, University of Amsterdam, 1098 XH Amsterdam, The Netherlands;; ^e^Institute for Advanced Studies, University of Amsterdam, 1012 GC Amsterdam, The Netherlands;; ^f^National Center of Cognitive Research, ITMO University, St. Petersburg Russia 199034

“Larger citiesprovide a buffer against depression”—this astounding statement is from a PNAS article by Stier et al. (1) on how depression rates scale with the population of metropolitan statistical areas (MSAs). It is astounding as it runs contrary to a wealth of psychological and epidemiological research showing the complex nature of depression and the detrimental influence of cities (2–4). This conflict with prior research makes it paramount to study the quality of the evidence. In this letter we carry out this task by considering the robustness of the finding. Our analysis suggests that their data do not support their conclusion.

Cities can be defined in a multitude of ways, so it is central for empirical research on cities to assess if findings are specific to the city definition used or whether they hold generally (5). Specifically for scaling studies it has been shown that their results depend heavily on minimum city size inclusion criteria and the spatial extent of cities (6). Stier et al. (1) address the former question using Behavioral Risk Factor Surveillance System data; here, their conclusion does not hold. When the minimum city size is lower than 600,000 inhabitants the sublinear scaling disappears. An additional concern comes from the fact that the original study does not assess the robustness under different spatial city definitions. We turn to this analysis next.

**Fig. 1.**
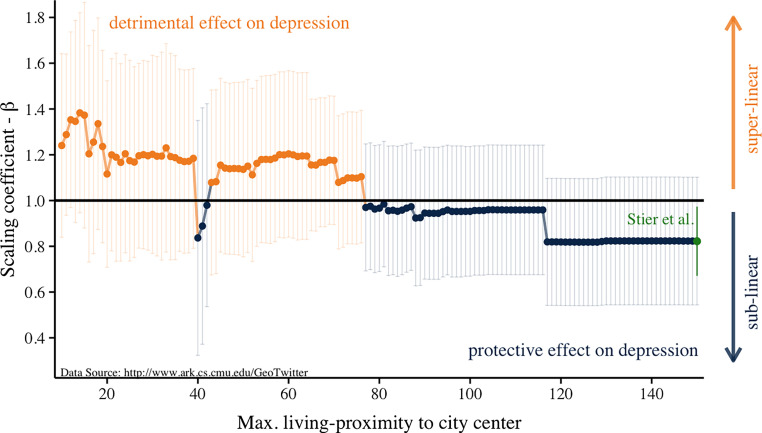
City effect on depression depends on living proximity to city center. Scaling analyses were conducted with varying samples depending on individuals’ proximity to city center using the Twitter 2010 data (7).

Our analysis utilizes the rich geographical information available in the Twitter 2010 dataset also used in the original study (7). We computed how the scaling coefficient changes with varying city sizes defined by the distance to city centers. The results are presented in Fig. 1. When including individuals living in an area with a radius larger than 78 km, we find a scaling coefficient below 1, replicating Stier et al.’s result that bigger cities are protective against depression (1). However, when using city boundaries with a smaller area, we find the opposite result: a scaling coefficient above 1. This supports the conclusion that larger cities increase the risk for depression.

Taken together, our robustness analysis shows that cities can have both a protective and a detrimental effect on depression depending on how they are defined. As we recall, Stier et al. (1) define cities as MSAs. These are large economically interrelated regions spanning urban, suburban, and rural areas. In fact, only 28.7% of MSA inhabitants report living in urban areas; 57.2% perceive their neighborhood as suburban, and 14.2% identify their surroundings as rural (8). This points to the same conclusion as the 78-km cutoff: The protective effect of cities on depression arises only when inner-city, suburban, and rural areas are combined. For this reason, we argue that the conclusion “larger cities provide a buffer against depression” is an unwarranted conclusion given the evidence.
